# Starch intake, amylase gene copy number variation, plasma proteins, and risk of cardiovascular disease and mortality

**DOI:** 10.1186/s12916-022-02706-5

**Published:** 2023-01-24

**Authors:** Huiping Li, Yan Borné, Yaogang Wang, Emily Sonestedt

**Affiliations:** 1 School of Public Health, Tianjin Medical University, 22 Qixiangtai Road, Heping District, Tianjin, 300070 China; 2 Nutritional Epidemiology, Department of Clinical Sciences in Malmö, Lund University, Malmö, Sweden

**Keywords:** Starch, *AMY1*, Cardiovascular disease, Mortality, Plasma proteins

## Abstract

**Background:**

Salivary amylase, encoded by the *AMY1* gene, initiate the digestion of starch. Whether starch intake or *AMY1* copy number is related to disease risk is currently rather unknown. The aim was to investigate the association between starch intake and *AMY1* copy number and risk of cardiovascular disease (CVD) and mortality and whether there is an interaction. In addition, we aim to identify CVD-related plasma proteins associated with starch intake and *AMY1* copy number.

**Methods:**

This prospective cohort study used data from 21,268 participants from the Malmö Diet and Cancer Study. Dietary data were collected through a modified diet history method and incident CVD and mortality were ascertained through registers. *AMY1* gene copy number was determined by droplet digital polymerase chain reaction, a risk score of 10 genetic variants in *AMY1* was measured, and a total of 88 selected CVD-related proteins were measured. Cox proportional hazards regression was used to analyze the associations of starch intake and *AMY1* copy number with disease risk. Linear regression was used to identify plasma proteins associated with starch intake and *AMY1* copy number.

**Results:**

Over a median of 23 years’ follow-up, 4443 individuals developed CVD event and 8125 died. After adjusting for potential confounders, a U-shape association between starch intake and risk of CVD (*P*-nonlinearity = 0.001) and all-cause mortality (*P*-nonlinearity = 0.03) was observed. No significant association was found between *AMY1* copy number and risk of CVD and mortality, and there were no interactions between starch intake and *AMY1* copy number (*P* interaction > 0.23). Among the 88 plasma proteins, adrenomedullin, interleukin-1 receptor antagonist protein, fatty acid-binding protein, leptin, and C-C motif chemokine 20 were associated with starch intake after adjusting for multiple testing.

**Conclusions:**

In this large prospective study among Swedish adults, a U-shaped association between starch intake and risk of CVD and all-cause mortality was found. Several plasma proteins were identified which might provide information on potential pathways for such association. *AMY1* copy number was not associated with CVD risk or any of the plasma proteins, and there was no interaction between starch intake and *AMY1* copy number on disease risk.

**Supplementary Information:**

The online version contains supplementary material available at 10.1186/s12916-022-02706-5.

## Background

Cardiovascular disease (CVD), a major cause of disability and premature death throughout the world, can be prevented to a large extent by a healthy diet [1]. Evidence on the role of carbohydrate intake on risk of CVD and mortality have been inconsistent [2–6], which could due to opposite effects by different types of carbohydrates. For example, sugar intake has been positively associated, while fiber intake has been inversely associated, with the various disease outcomes [7, 8]. Compared with sugar and fiber intake, the association between starch intake and risk of CVD and mortality have been investigated to a limited extent and the results are inconsistent [9–11]. The inconsistency could be due to genetic variation across populations.

Starch digestion is initiated in the oral cavity by the action of salivary amylase, which is encoded by the salivary amylase gene (*AMY1*) [12]. Although foods remain in the mouth for a relatively short time, salivary amylase bound to starch can also be detected in the duodenum [13]. The copy number variation (CNV) of the *AMY1* gene, ranging from 2 to 17 diploid copies, is correlated to salivary amylase concentrations and was found to be higher in populations that historically have consumed large amounts of starch [12, 14]. A modestly higher postprandial glycemia after a starchy meal has been shown among individuals with high *AMY1* copy number compared to those with low *AMY1* copy number [15, 16], although some studies found no difference [17, 18]. In addition, the association between *AMY1* copy number and metabolic traits is inconsistent [17, 19–21], although a negative association between *AMY1* copy number and several cardiometabolic risk factors, including obesity and inflammation which have been shown in some studies, mainly will be small sample sizes [22–24]. Thus, there is a need to further explore the association between *AMY1* copy number and cardiometabolic risk markers, especially to simultaneously examine a broad range of risk markers, including inflammatory markers. In addition, to the best of our knowledge, there are no studies that have investigated the association between *AMY1* copy number and risk of CVD and mortality. Although we did not find significant associations between *AMY1* copy number and body mass index (BMI) or glucose homeostasis traits in our previous study, we observed an interaction between *AMY1* copy number and starch intake on BMI and insulin resistance [20, 21]. For example, the inverse association of high-starch diet and fasting insulin levels was stronger with higher *AMY1* copy numbers [20].

Therefore, the aim of this study was to investigate the association between starch intake, *AMY1* copy number, and risk of CVD and mortality and to examine the interaction between starch intake and *AMY1* copy number. In addition, we aim to identify whether any of 88 CVD-related plasma proteins were associated with starch intake or *AMY1* copy number.

## Methods

### Study population

The Malmö Diet and Cancer Study (MDCS) is a population-based prospective cohort study initiated to examine associations between diet and health outcomes [25]. It includes 30,446 individuals from Malmö, in the south of Sweden, aged 44–74 years at enrollment between 1991 and 1996. At baseline, all the MDCS participants underwent physical examinations, had blood taken, and answered questionnaires regarding lifestyle factors. Between October 1991 and February 1994, 6103 individuals was randomly selected to be studied for the epidemiology of carotid artery disease, and this sample is referred to as the Malmö Diet and Cancer-Cardiovascular Cohort (MDC-CC) [26]. All participants provided written informed consent. The study was approved by the Ethical Review Board at the Faculty of Medicine at Lund University, Sweden (LU 51/90), and was carried out in accordance with the Helsinki Declaration.

Among the 30,446 participants, we excluded those with missing information on dietary intake (*n* = 2213) and other relevant covariates (*n* = 563). We also excluded participants with a history of CVD (*n* = 829), diabetes (*n* = 1123), and cancer (*n* = 1574) at baseline, leaving 24,144 individuals. To reduce the bias of population stratification for the genetic analyses, we further excluded participants who were not born in Sweden or with unknown country of birth (*n* = 2876), leaving 21,268 individuals. A total of 20,264 individuals had available data on specific genetic variants within the *AMY1* gene, and 3932 had information on *AMY1* copy number.

### Dietary assessment and calculation of starch intake

Dietary intake was assessed using a modified diet history method consisting of (1) a 7-day food diary covering all cooked meals, cold beverages, and dietary supplements; (2) a 168-item food-frequency questionnaire (FFQ) for assessment of consumption frequencies and portion sizes of regularly eaten foods that were not covered by the 7-day food diary; and (3) an interview to ask for cooking methods and usual portion sizes for foods recorded in the food diary and to check for overlap between intakes reported by the food diary and the FFQ. Daily food intake was calculated by combing the intake data from the FFQ and the food diary. The energy and nutrient intake values were obtained from the MDCS Food and Nutrient Database that originated from PC KOST2-93, based on the Swedish National Food Agency. Starch intake was calculated by subtracting the intake of total sugars (i.e., mono- and disaccharides) from the total carbohydrate intake and was expressed as the percentage of total non-alcoholic energy intake (E%). The participants were divided into sex-specific quartiles based on their starch intake. Data on the validity [27, 28] and reproducibility [29] of the method have been published. The energy-adjusted Pearson correlation coefficients for carbohydrate intake were 0.66 and 0.70 for men and women, respectively.

### Genotyping

*AMY1* gene copy number was determined by droplet digital polymerase chain reaction (ddPCR) using the QX200 AutoDG ddPCR System (Bio-Rad Laboratories, Hercules, CA) following the manufacturer’s instructions, as previously described in detail by Rukh et al. [21]. Samples were analyzed along with negative control (RNase free water) on each plate. A high copy number control (NA18972; HapMap DNA: 17 copies of *AMY1*) and 2 samples from female donors with known *AMY1* copy numbers (4 and 8 copies of *AMY1*) were included as controls in each run. Samples with high copy numbers were diluted and reanalyzed to exclude the possibility of the presence of too much DNA or a technical error. After amplification, the reactions were stored at 4 °C until the plates were read on a Bio-Rad QX200 Droplet Reader, and copy numbers were calculated through the use of the QuantaSoft software version 1.7.4 (Bio-Rad Laboratories). Copy number of *AMY1* was divided into four groups: 1–4, 5–6, 7–9, and 10 and above copies.

We created *AMY1*-genetic risk scores (GRS) using 10 single nucleotide polymorphisms (SNPs) associated with copy number variation in *AMY1* gene loci based on a previous publication of European data [30]. The relationship of these SNPs to *AMY1* copy number was consistent across 2 independent cohorts of European-ancestry individuals sampled in the USA and Europe [30]. Individual SNP was coded as 0, 1, and 2 according to the number of increasing alleles. We calculated a weighted GRS using the following equation: *AMY1*-GRS = (*β*1 × SNP1 + *β*2 × SNP2 +…+ *β*3 × SNP3) × (*n*/sum of the *β* coefficients), where *β* is the *β* coefficient of each SNP for change in *AMY1* copy number in the MDCS, and *n* was 10. The spearman correlation coefficient between *AMY1* copy number and *AMY1*-GRS is 0.43 in our cohort. Details of SNPs and *β* coefficients of each SNP are presented in Additional file 1: Table S1.

### Plasma protein quantification

For plasma proteins, the analysis was restricted to the MDC-CC participants. The available sample was 3680 and 3254 participants for identifying the proteins associated with starch intake and *AMY1* copy number, respectively. Serum samples were stored frozen until proteomic profiling was performed using the Olink platform (Olink Proteomics, Uppsala, Sweden) (https://www.olink.com/content/uploads/2015/12/0696-v1.3-Proseek-Multiplex-CVD-I-Validation-Data_final.pdf). A total of 92 plasma proteins were measured by the SciLifeLab with the Olink Proseek Multiplex proximity extension assays Cardiovascular. Values were expressed as normalized protein expression values as arbitrary units on a log2 scale. For statistical analysis, we excluded four proteins that were available in less than 75% of the individuals in the present study sample. Hence, 88 proteins were available for the analysis.

### Outcome

All participants were followed up from baseline examination to the diagnosis of CVD, emigration from Sweden, death, or December 31, 2018, whichever came first. Total CVD and mortality were obtained through the Swedish Hospital Discharge Register [31], the Swedish Cause of Death Register, and the Stroke Register of Malmö [32]. Total CVD was defined as a hospital admission or death using International Classification of Diseases (ICD) codes, including coronary heart disease (ICD-9 codes 410A-410X and ICD-10 code I21), death attributable to ischemic heart disease (ICD-9 codes: 410-414; ICD-10 codes: I20-I25), and ischemic stroke (ICD-9 code 434 and ICD-10 code I63). CVD mortality was defined as ICD-9 codes 390-459 and ICD-10 I00-I99.

### Assessment of covariates

Data on smoking habits (current, former, or never), educational level (the highest qualification attained), and heredity for diseases (including cancer, myocardial infarction, stroke, and diabetes) and other lifestyle factors were obtained using a structured questionnaire. Four separate heredity scores were constructed and participants were categorized as having low heredity (e.g., with no first-degree relatives with the disease), medium heredity (with one first-degree relative with the disease), and high heredity (with at least two first-degree relatives with the disease) for disease [33]. Alcohol consumption was divided into six categories. Zero consumers reported no consumption of alcohol in the 7-day food diary or during the previous year in the questionnaire. The other individuals were divided into sex-specific quintiles based on the reported alcohol intake from the 7-day food diary. Leisure-time physical activity was categorized into five predefined groups based on metabolic equivalent task (MET) hours per week: < 7.5, 7.5–15, 15–25, 25–50, and > 50 MET hour/week. BMI (kg/m^2^) was calculated as weight in kilograms divided by the square of the height in meters. Systolic and diastolic blood pressures were measured after 10 min of rest in the supine position. Hypertension was defined as systolic/diastolic blood pressures ≥ 140/90 mmHg and/or current use of antihypertensive medications. The variable for dietary assessment method was created based on the interview time of 60 min before September 1994 and 45 min from 1 September 1994. The season of diet data collection was defined as spring, summer, autumn, and winter. A diet quality index based on the Swedish dietary guidelines was calculated using the following factors: fiber (> 2.4 g/MJ), fruit and vegetables (> 400 g/day), fish (> 300 g/week), added sugar (< 10% energy), and red and processed meat (< 500 g/week). Each point was given for each favorable diet factor, with the total score ranging from 0 to 5 [34].

### Statistical analysis

To evaluate differences in baseline characteristics for participants with different quartiles of starch intake, 𝜒2 test was conducted for categorical variables, and analysis of variance was conducted for continuous variables. Cox proportional hazards models were conducted with quartiles of sex-specific starch intake as the exposures of interest, incident CVD and mortality as the outcome, and time-to-event as the time metric. We tested for linear trends by using the ordinal score on quartiles of starch intake. Three models were applied in our analyses. Model 1 was adjusted for age, sex, dietary assessment method, season, and total energy intake. Model 2 was additionally adjusted for leisure-time physical activity, smoking status, alcohol consumption, education, heredity score, and diet quality index. Model 3 was further adjusted for hypertension and BMI. Interaction by cereal intake, bread intake, potato intake, and sex were performed by adding a multiplicative factor between these starch food source (quartiles) and starch (continues) in model 3.

Age- and sex-adjusted Cox proportional hazards models were used to examine the associations of *AMY1* copy number and *AMY1*-GRS with risk of CVD and mortality. Interaction by age were performed by adding a multiplicative factor between age and *AMY1* copy number in age- and sex-adjusted model. Furthermore, multiplicative interactions between *AMY1* copy number or *AMY1*-GRS and starch intake were evaluated using the Wald test on cross-product terms into multivariable models (the above model 3). The association between starch intake and risk of CVD and mortality were evaluated in strata of *AMY1* copy number (1–4 copies, 5–6 copies 7–9 copies, 10 and above copies) and *AMY1*-GRS (quintiles). We verified the assumption of linearity between starch intake, *AMY1* copy number, *AMY1*-GRS, and risk of CVD and mortality using restricted cubic spline functions with the SAS macro written by Desquilbet and Mariotti [35].

Associations between starch intake and plasma proteins were studied with three multivariable linear regression models: model 1 adjusted for age, sex, season, and total energy intake, model 2 with further adjustment for education, smoking status, alcohol consumption, and leisure-time physical activity, and model 3 with additional adjustment for BMI. Associations between *AMY1* copy number and plasma proteins were studied with multivariable linear regression, adjusted for age and sex. Tests of statistical significance were corrected for multiple comparisons using the Bonferroni method for 88 tests (*P* < 0.00057).

Cox proportional hazards models were used to study associations between proteins that were associated with starch intake or *AMY1* copy number (after Bonferroni correction) and risks of CVD and mortality. Models 1 were adjusted for age and sex, and model 2 were further adjusted for physical activity, smoking status, alcohol consumption, educational level, and heredity scores. To test for the potential influence of obesity in the association between plasma protein and risk of CVD and mortality, we additionally adjusted this model for BMI (model 3). All tests were two-sided, and we considered *P* < 0.05 to be statistically significant. SAS version 9.4 (SAS Institute Inc., Cary, NC, USA) was used for the analyses.

## Results

A total of 21,268 participants (8187 (38.5%) men and 13,081 (61.5%) women) were included in the study. At baseline, the mean intake of starch in the total population was 24E% or 134 g/day.

Table 1 shows the baseline characteristics of participants according to quartiles of sex-specific starch intake. Compared with participants in the lowest quartile, participants in the highest quartile of starch intake tended to be younger, more often ex-smokers and non-smokers, and less likely to have hypertension, but were more likely to have family history of myocardial infarction and diabetes. They had lower intake of energy and fat and higher intake of carbohydrates, protein, potato, and bread and higher diet quality score.

**Table 1:** Baseline characteristics of the participants according to sex specific quartiles of starch intake (*n* = 21,268)

Characteristics	Quartiles of starch intake (E%)				*P* for trend^a^
	1 (*n* = 5318)	2 (*n* = 5317)	3 (*n* = 5315)	4 (*n* = 5318)	
Starch intake (g/day)	112.9 ± 35.2	127.3 ± 36.8	138.4 ± 41.5	156 ± 50.8	< 0.001
Starch intake (E%)	19.2 ± 1.9	22.8 ± 1.0	25.4 ± 1.1	29.5 ± 2.8	< 0.001
Age (years)	58.3 ± 7.5	58.1 ± 7.6	57.4 ± 7.7	57.2 ± 7.5	< 0.001
Sex (men, %)	38.5	38.5	38.5	38.5	1.00
BMI (kg/m^2^)	25.5 ± 4	25.7 ± 3.8	25.5 ± 3.9	25.2 ± 3.7	0.7200
SBP (mmHg)	141 ± 19.6	141 ± 19.5	140.3 ± 20	140.1 ± 19.9	0.08
DBP (mmHg)	85.9 ± 9.8	85.6 ± 9.9	85.3 ± 9.9	85.2 ± 10	0.00
Total energy intake (kcal/day)	2422.8 ± 700.9	2304.5 ± 622.7	2253.4 ± 623.3	2185.7 ± 622.7	< 0.001
LTPA (> 25 MET-hour/week, %)	51.9	53.2	53.57	53.08	0.19
Zero-consumers of alcohol (%)	6.56	4.95	4.48	5.57	0.01
University degree (%)	14.5	14.2	15.0	14.7	0.49
Smoking status (%)					
Smoker	37.9	27.8	24.9	22.4	< 0.001
Ex-smoker	29.4	32.9	34.6	35.4	< 0.001
Non-smoker	32.7	39.4	40.5	42.2	< 0.001
Hypertension (%)	61.0	60.7	59.1	58.8	< 0.01
Heredity score (> 0, %)					
Cancer	47.2	47.6	45.8	45.7	0.04
Myocardial infarction	36.6	38.0	37.8	39.3	0.01
Stroke	27.5	26.2	26.7	25.9	0.12
Diabetes	1.69	1.66	2.14	2.20	0.02
Diet quality index	1.6 ± 1.2	1.8 ± 1.3	1.9 ± 1.3	2.3 ± 1.3	< 0.001
Carbohydrate (E%)	41.2 ± 5.9	43.9 ± 5	45.8 ± 4.8	49.0 ± 5.2	< 0.001
Protein (E%)	15.6 ± 2.9	15.7 ± 2.4	15.8 ± 2.4	15.8 ± 2.3	< 0.001
Fat (E%)	43.2 ± 5.9	40.4 ± 5.1	38.3 ± 4.8	35.2 ± 5.1	< 0.001
Potato (g/day)	111.3 ± 64.5	120.2 ± 66.7	127.9 ± 75.7	133.8 ± 85.7	< 0.001
Bread (g/day)	88.6 ± 48.1	104.8 ± 55.7	117.5 ± 64.6	148.3 ± 91.5	< .0001

Continuous variable are expressed as means ± standard deviations and categorical variables are expressed as percentages

*BMI* body mass index, *DBP* diastolic blood pressure, *LTPA* leisure-time physical activity, *MET* metabolic equivalent, *SBP* systolic blood pressure

^a^General linear models or logistic regression analysis. The *P* values for trend were calculated by using sex specific quartiles of starch intake as an ordinal variable

**Fig. 1:**
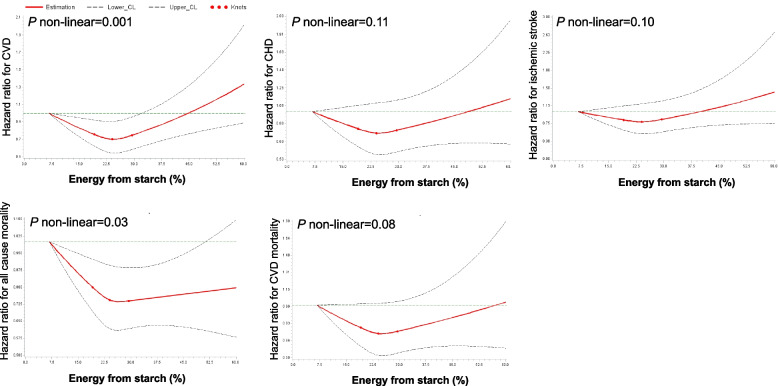
Restricted cubic spline plots to assess association between starch intake and CVD and mortality. The HRs and 95% CIs above were adjusted for age, sex, smoking status, drinking status, education, season, method, physical activity, heredity scores (including cancer, infarct, stroke, and diabetes), total energy intake, the modified diet index, hypertension, and body mass index. CVD, cardiovascular disease; CHD, coronary heart disease

During median follow-up of 23 years, 4443 (20.9%) of the participants had a cardiovascular event and 8125 (38.2%) died. Table 2 shows the association between starch intake and the risk of CVD and mortality. The restricted cubic spline analyses indicated U-shape associations between starch intake and CVD risk (*P*-nonlinearity = 0.0012) and all-cause mortality (*P*-nonlinearity = 0.03) (Fig. 1). Compared with those in the lowest quartile, those in the second to the third quartile had a 9–11% lower risk of CVD, while there was no statistically reduced risk in the highest quartile (Table 2). Participants in the highest vs. lowest quartile had a hazard ratio of 0.91 (95% confidence interval 0.85 to 0.97) for all-cause mortality. Similar non-linear associations (*P*-nonlinearity = 0.08–0.11), although not statistically significant were observed between starch intake and CHD, ischemic stroke, and CVD mortality (Fig. 1). There was no heterogeneity of effect for cereal intake, bread intake, potato intake, and sex on association of starch intake and CVD (all *P*-interaction > 0.07) and mortality risk (all *P*-interaction > 0.07).

**Table 2:** Associations between intake of starch and CVD and mortality risk (*n* = 21,268)

	Quartiles of starch consumption				*P* trend	Continuous^a^	*P* value
	Quartile 1	Quartile 2	Quartile 3	Quartile 4			
**Starch (E%, median)**	19.6	22.8	25.3	28.8			
**CVD**							
Cases/non-case	1236/4082	1108/4209	1022/4293	1077/4241	-	-	-
Model 1^b^	1 [reference]	0.86 (0.79, 0.93)	0.81 (0.74, 0.88)	0.86 (0.79, 0.93)	< 0.001	0.89 (0.82, 0.95)	< 0.01
Model 2^c^	1 [reference]	0.91 (0.84, 0.99)	0.88 (0.81, 0.95)	0.95 (0.87, 1.03)	0.14	0.98 (0.91, 1.05)	0.51
Model 3^d^	1 [reference]	0.91 (0.84, 0.99)	0.89 (0.82, 0.97)	0.98 (0.90, 1.07)	0.49	1.00 (0.93, 1.08)	0.96
**CHD**							
Cases/non-case	717/4601	601/4716	591/4724	612/4706	-	-	-
Model 1	1 [reference]	0.81 (0.72, 0.90)	0.81 (0.73, 0.90)	0.84 (0.75, 0.94)	< 0.01	0.85 (0.78, 0.94)	< 0.01
Model 2	1 [reference]	0.86 (0.77, 0.96)	0.89 (0.80, 1.00)	0.94 (0.84, 1.06)	0.37	0.95 (0.87, 1.05)	0.34
Model 3	1 [reference]	0.87 (0.78, 0.97)	0.91 (0.81, 1.01)	0.99 (0.88, 1.10)	0.88	0.99 (0.90, 1.09)	0.79
**Ischemic stroke**							
Cases/non-case	512/4806	512/4805	427/4888	441/4877	-	-	-
Model 1	1 [reference]	0.96 (0.85, 1.09)	0.83 (0.73, 0.95)	0.86 (0.76, 0.98)	< 0.01	0.93 (0.83, 1.03)	0.17
Model 2	1 [reference]	1.01 (0.89, 1.14)	0.89 (0.78, 1.01)	0.94 (0.82, 1.07)	0.13	0.98 (0.91, 1.05)	0.51
Model 3	1 [reference]	1.01 (0.89, 1.14)	0.90 (0.79, 1.02)	0.96 (0.84, 1.10)	0.27	1.03 (0.91, 1.15)	0.67
**All-cause mortality**							
Cases/non-case	2315/3003	2027/3290	1930/3385	1853/3465			
Model 1	1 [reference]	0.83 (0.79, 0.88)	0.83 (0.78, 0.88)	0.80 (0.75, 0.85)	< 0.001	0.82 (0.77, 0.86)	< 0.001
Model 2	1 [reference]	0.90 (0.85, 0.96)	0.92 (0.86, 0.98)	0.90 (0.84, 0.96)	< 0.01	0.92 (0.87, 0.97)	< 0.01
Model 3	1 [reference]	0.90 (0.85, 0.95)	0.92 (0.87, 0.98)	0.91 (0.85, 0.97)	0.01	0.93 (0.88, 0.98)	< 0.01
**CVD mortality**							
Cases/non-case	742/4576	610/4707	611/4704	592/4726			
Model 1	1 [reference]	0.78 (0.70, 0.87)	0.83 (0.74, 0.92)	0.81 (0.72, 0.90)	< 0.001	0.83 (0.76, 0.92)	< 0.01
Model 2	1 [reference]	0.84 (0.76, 0.94)	0.92 (0.82, 1.02)	0.91 (0.81, 1.02)	0.22	0.94 (0.85, 1.03)	0.19
Model 3	1 [reference]	0.84 (0.75, 0.94)	0.93 (0.84, 1.04)	0.94 (0.84, 1.05)	0.22	0.96 (0.87, 1.06)	0.43

Obtained by using multivariable Cox regression model

^a^Hazard ratio for per increase of 10% in proportion of starch energy in total energy

^b^Model 1 was adjusted for age and sex

^c^Model 2 was additionally adjusted for smoking status, drinking status, education, season, method, physical activity, heredity scores (including cancer, infarct, stroke, and diabetes), total energy intake, and the modified diet index

^d^Model 3 was for adjusted the same variables as in model 3 and further for HBP and BMI

**Fig. 2:**
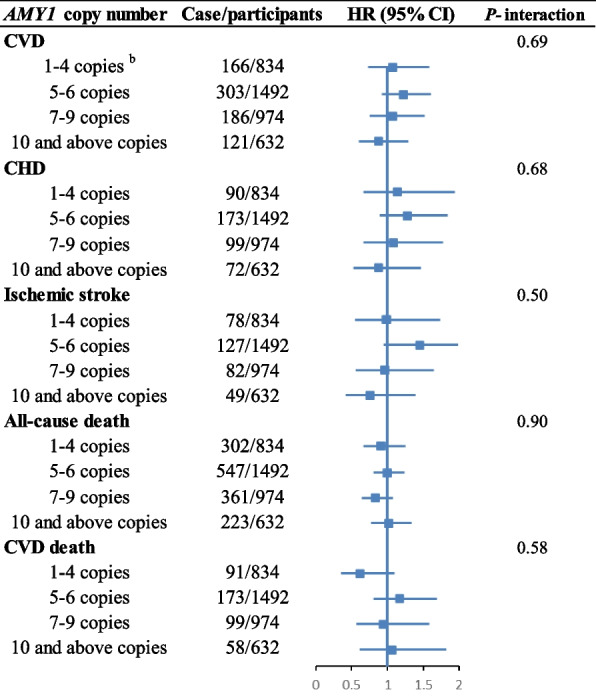
Association between intake of starch and risk of CVD and mortality by *AMY1* copy number^a^. ^a^The multivariable analysis was adjusted for age, sex, smoking status, drinking status, education, season, method, physical activity, heredity scores (including cancer, infarct, stroke, and diabetes), total energy intake, the modified diet index, hypertension, and body mass index. ^b^Hazard ratio for per increase of 10% in proportion of starch energy in total energy. CVD, cardiovascular disease; CHD, coronary heart disease; CI, confidence interval; HR, hazards ratio

We did not observe statistically significant associations between *AMY1* copy number and any of the outcomes (Table 3 and Additional file 1: Fig. S2). There was no heterogeneity of effect for age on association of *AMY1* copy number and CVD (*P*-interaction = 0.07) and mortality risk (*P*-interaction = 0.14). In addition, no significant associations were found between *AMY1*-GRS and any of the outcomes (Additional file 1: Table S2 and Fig. S3). There was no evidence of interaction between starch intake and *AMY1* copy number or *AMY1*-GRS on CVD and mortality risk (Fig. 2 and Additional file 1: Fig. S4). Neither *AMY1* copy number (*P* = 0.58) nor *AMY1*-GRS (*P* = 0.85) were associated with starch intake.

**Table 3:** Associations between intake of *AMY1* copy number and CVD and mortality risk (*n* = 3932)

	*AMY1* copy number				*P* trend	Continuous^a^	*P* value
	1–4 copies	5–6 copies	7–9 copies	10 and above copies			
**CVD**							
Cases/non-case	166/668	303/1189	186/788	121/511	-		
Age- and sex-adjusted model	1 [reference]	1.03 (0.86, 1.25)	0.98 (0.79, 1.20)	0.99 (0.78, 1.25)	0.75	1.02 (0.99, 1.05)	0.27
**CHD**							
Cases/non-case	90/744	173/1319	99/875	72/560	-		
Age- and sex-adjusted model	1 [reference]	1.10 (0.85, 1.42)	0.96 (0.72, 1.27)	1.09 (0.80, 1.48)	0.95	1.03 (0.99, 1.07)	0.17
**Ischemic stroke**							
Cases/non-case	78/756	127/1365	82/892	49/583	-		
Age- and sex-adjusted model	1 [reference]	0.93 (0.70, 1.23)	0.92 (0.67, 1.25)	0.83 (0.58, 1.19)	0.33	1.01 (0.96, 1.06)	0.76
**All-cause mortality**							
Cases/non-case	302/532	547/945	361/613	223/409			
Age- and sex-adjusted model	1 [reference]	1.03 (0.90, 1.19)	1.04 (0.89, 1.21)	0.96 (0.81, 1.15)	0.77	1.01 (0.99, 1.03)	0.49
**CVD mortality**							
Cases/non-case	91/743	173/1319	99/875	58/574			
Age- and sex-adjusted model	1 [reference]	1.08 (0.84, 1.39)	0.94 (0.71, 1.25)	0.83 (0.60, 1.15)	0.16	1.02 (0.98, 1.06)	0.44

Obtained by using a multivariable logistic regression model

^a^Hazard ratio for per increase of 1 *AMY1* copy number

**Fig. 3:**
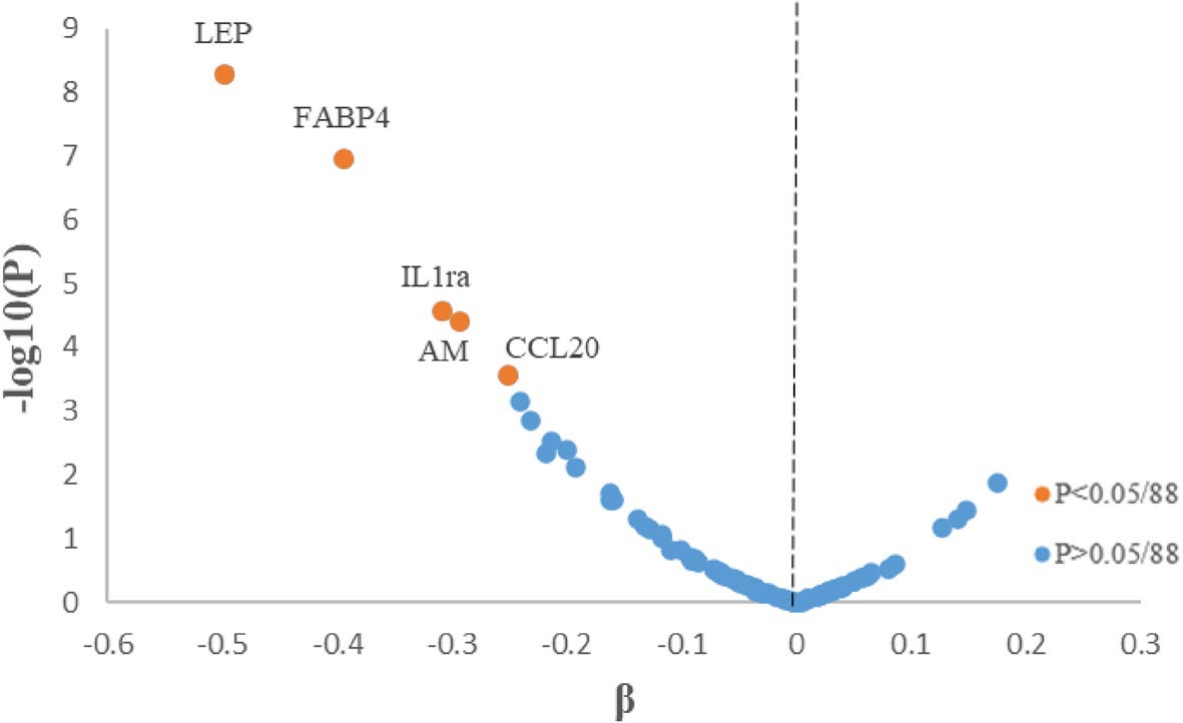
Volcano plot of association between starch intake and 88 plasma proteins in full sample analysis (*n* = 3680). Linear regressions were adjusted for age, sex, season, total energy intake, educational level, smoking status, alcohol consumption, and physical activity

After adjustment for potential confounders and correction for multiple testing, concentrations of the following five among the 88 plasma proteins were found to be significantly and inversely associated with starch intake: adrenomedullin, interleukin-1 receptor antagonist protein (IL1ra), fatty acid-binding protein (FABP4), leptin, and C-C motif chemokine 20 (CCL20) (Fig. 3). The associations were attenuated and not statistically significant after further adjustment for BMI (Additional file 1: Table S3). All five starch-related proteins were positively associated with risk of CVD, CHD, and all-cause mortality in model 2, adjusting for lifestyle factors (*P* < 0.05). Higher levels of leptin, IL1ra, and adrenomedullin were associated with increased ischemic stroke risk. Higher levels of leptin, FABP4, and IL1ra were associated with increased CVD mortality. Overall, the associations were not significant after further adjustment for BMI (Additional file 1: Table S4). None of the plasma proteins were associated with the *AMY1* copy number with adjustment for age and sex (Additional file 1: Table S5).

## Discussion

In this large prospective cohort of Swedish middle aged and elderly individuals, we found non-linear associations between starch intake and risk of CVD and all-cause mortality. We identified five plasma proteins linked to lower starch intake that were also associated with increased CVD and mortality risk, providing clues to underlying biological mechanisms. *AMY1* copy number was not associated with plasma proteins or CVD and mortality risk, and we did not observe any interaction between *AMY1* copy number and starch intake on risk of CVD and mortality.

The observed U-shaped associations between starch intake and risk of CVD and all-cause mortality observed in our study was similar to previous results in the prospective UK Biobank cohort, which reported lowest risk of CVD and death among participants consuming 20–30E% from starch [10]. In contrast, a prospective cohort study conducted in Japan reported an inverse association between starch intake and all-cause mortality in men [11]. These studies, including ours, have adjusted for total energy intake in the analyses and thereby examining the isocaloric exchange of starch with other macronutrients. This discrepancy might be due to the different levels and sources of starch in Japan and Europe. For example, the mean intake of starch is approximately 39E% in the Japanese study and 24E% both in the UK Biobank study and our study. Furthermore, the main source of starch in Japan is rice, while in Europe, it is potato and bread. In a meta-analysis, high rice consumption was related to a modest reduction in risk of mortality in men [36]. The mean intake of starch in our population was 134 g/day, which is approximately equal to the starch content in 300 g of bread or 1 kg of potatoes [37]. Previous studies have shown a protective association of total bread with mortality [38]. However, a recent meta-analysis did not find a significant association between potato consumption and risk of all-cause mortality [39]. In addition, although not statistically significant, a U-shaped association was also found between starch intake and risk of CHD and stroke in our study. Similarly, previous prospective studies have not observed the association between starch intake and risk of CHD among US, Chinese, and European populations [9, 40–42] and risk of stroke among Europeans [43].

We identified adrenomedullin, IL1ra, FABP4, leptin, and CCL20 to be inversely associated with starch intake, and all these proteins were positively associated with increased risk of CVD and mortality. Plasma FABP4, leptin, IL1ra, and CCL20 play a regulatory role in inflammation [44, 45], which is an underlying mechanism of CVD and death. Furthermore, adrenomedullin, leptin, and FABP4 are secreted by the adipose tissue in direct relation to the amount of body fat [46–48], and IL1RA and CCL20 has been shown to be highly elevated in obese animals and humans [49–51]. Animal experiments and human trials showed that resistant starch (i.e., starch that is not digested and absorbed in the small intestine) had an anti-inflammatory effect [52]. In addition, several studies have shown that higher intakes of slowly digestible starch and resistant starch intake are associated with increased satiety, reduced hunger, and/or reduced body weight [53, 54]. Although it is not easy to explain the association with starch, high intakes may inhibited the expression and function of plasma proteins by increasing insulin resistance and plasma-free fatty acids [55], as well as delaying adipocyte differentiation [56].

In the present study, *AMY1* copy number was not associated with any of the outcomes, and as far as we know, there are no previous study that has investigated this. *AMY1* copy number was only measured in 3932 of the participants and *AMY1*-GRS was measured in 20,264 of the participants. Thus, the size of our cohort may have been underpowered to detect small differences and future study needs to confirm such association in other populations. Because several previous studies showed no or weak associations with CVD risk factors (e.g., inflammation, obesity, and insulin sensitivity) [22, 24, 57], we might not expect any major association of *AMY1* copy number and CVD risk.

We did not identify any of the included plasma proteins, including several inflammatory markers, to be associated with *AMY1* copy number after correction for multiple testing. To our knowledge, the current study is the first to examine a broad range of CVD-related plasma proteins, including inflammatory markers (e.g., interleukin (IL), tumor necrosis factor, and CCL) associated with *AMY1* copy number. A previous study of 57 non-diabetic adults found that individuals with low *AMY1* CNV had higher concentrations of serum cytokines, including IL-6, IL-1β, monocyte chemoattractant protein-1, and tumor necrosis factor-alpha [22]. Another study of 76 children found that *AMY1* CNV were negatively associated with the C-reactive protein, resistin, and CCL2 [58].

The strengths of the study include its prospective study design, long follow-up time, detailed dietary assessment method, and more precise measurement of *AMY1* copy number using ddPCR. Nevertheless, several limitations must be acknowledged. Firstly, dietary intakes were collected only at baseline and the dietary habits may have changed over time, which could have potentially underestimated the diet-disease associations. Second, our study only includes individuals of European descent. Thus, caution should be taken when generalizing the findings to other populations. In particular, the consumption of starch could be substantially different in other regions, and there might be diversity in the distribution of genes across different populations. Third, the lack of replication in external cohorts of the plasma proteins in relation to starch intake is a potential concern. Fourth, we were unable to investigate associations with different structures of starch because these data were not available, which could be an effect modifier. Therefore, future studies should focus on the influence of different types of starch on CVD and mortality. Finally, as in other observational studies, even though we have adjusted for a wide variety of covariates that relate to CVD and mortality, residual confounding by unidentified confounders is still possible.

## Conclusions

In conclusion, we observed U-shaped associations of starch intake with risk of CVD and mortality, and the plasma proteomic analysis which identified several plasma proteins associated with starch intake and CVD risk could provide clues to underlying biological mechanisms. *AMY1* copy number was not associated with risk of CVD, mortality, or CVD-related plasma proteins, and there was no interaction between starch intake and *AMY1* copy number.

## Supplementary Information

**Additional file 1: Table S1.** A total of 10 single nucleotide polymorphisms associated with *AMY1* copy number. **Table S2.** Association between intake of *AMY1*-GRS and CVD and mortality risk (*n* = 20,264). **Table S3.** Association between starch intake and plasma proteins (*n* = 3680). **Table S4.** Association between plasma proteins and CVD and mortality risk (*n* = 3680). **Table S5.** Association between *AMY1* copy number and plasma proteins (*n* = 3254). **Figure S1.** Flowchart of participant selection from the Malmö Diet and Cancer Study. **Figure S2.** Restricted cubic spline plots to assess association between *AMY1* copy number and CVD and mortality. **Figure S3.** Restricted cubic spline plots to assess association between *AMY1*-GRS and CVD and mortality. **Figure S4.** Association between intake of starch and risk of CVD and mortality by quintiles of *AMY1*-GRS (*n* = 20,264).

## Data Availability

The data that support the findings of this study are available from the Malmö Population-Based Cohorts Joint Database but restrictions apply to the availability of these data, which were used under license for the current study, and so are not publicly available. Data are however available from the authors upon reasonable request and with permission of the Malmö Population-Based Cohorts Joint Database.

## Abbreviations

BMI: Body mass index
CCL20: C-C motif chemokine 20
CNV: Copy number variation
CVD: Cardiovascular disease
E%: Percentage of total non-alcoholic energy intake
FABP4: Fatty acid-binding protein
FFQ: Food frequency questionnaire
GRS: Genetic risk score
ICD: International Classification of Diseases
IL1ra: Interleukin-1 receptor antagonist protein
IL: Interleukin
MDCS: The Malmö Diet and Cancer Study
MDC-CC: The Malmö Diet and Cancer-Cardiovascular Cohort
MET: Metabolic equivalent task
SNPs: Single nucleotide polymorphisms
