# Supplementary material for: Starch intake, amylase gene copy number variation, plasma proteins, and risk of cardiovascular disease and mortality
Source: BMC Med. 2023 Jan 24;21:27. doi: 10.1186/s12916-022-02706-5 (PMC9872432; doi:10.1186/s12916-022-02706-5)
Supplement: Supplementary file 1 — Additional file 1: Table S1. A total of 10 single nucleotide polymorphisms associated with AMY1 copy number. Table S2. Association between intake of AMY1-GRS and CVD and mortality risk (n = 20,264). Table S3. Association between starch intake and plasma proteins (n = 3680). Table S4. Association between plasma proteins and CVD and mortality risk (n = 3680). Table S5. Association between AMY1 copy number and plasma proteins (n = 3254). Figure S1. Flowchart of participant selection from the Malmö Diet and Cancer Study. Figure S2. Restricted cubic spline plots to assess association between AMY1 copy number and CVD and mortality. Figure S3. Restricted cubic spline plots to assess association between AMY1-GRS and CVD and mortality. Figure S4. Association between intake of starch and risk of CVD and mortality by quintiles of AMY1-GRS (n = 20,264). [file 12916_2022_2706_MOESM1_ESM.docx]

**Additional File 1**

**Table S1.** A total of 10 single nucleotide polymorphisms associated with *AMY1* copy number.

**Table S2.** Association between intake of *AMY1*-GRS and CVD and mortality risk (n=20,264).

**Table S3.** Association between starch intake and plasma proteins (n=3,680).

**Table S4.** Association between plasma protein and CVD and mortality risk (n=3,680).

**Table S5.** Association between *AMY1* copy number and plasma proteins (n=3,254).

**Fig. S1.** Flowchart of participant selection from the Malmö Diet and Cancer Study.

**Fig. S2.** Restricted cubic spline plots to assess association between *AMY1* copy number and CVD and mortality.

**Fig. S3.** Restricted cubic spline plots to assess association between *AMY1*-GRS and CVD and mortality.

**Fig. S4.** Association between intake of starch and risk of CVD and mortality by quintiles of *AMY1*-GRS (n=20,264).

| **Table S1.** A total of 10 single nucleotide polymorphisms associated with *AMY1* copy number. | | | | |
| --- | --- | --- | --- | --- |
| RS number | Effect Allele (EA) for  increases in CNVs | Beta | SE | *P* value |
| rs4244372 | T | 1.21 | 0.05 | <10^-6^ |
| rs11577390 | T | 2.24 | 0.10 | <10^-6^ |
| rs1566154 | G | 0.65 | 0.07 | <10^-6^ |
| rs1930212 | A | 0.72 | 0.07 | <10^-6^ |
| rs10881197 | A | 0.62 | 0.06 | <10^-6^ |
| rs2132957 | A | 1.41 | 0.14 | <10^-6^ |
| rs11185098 | A | 0.98 | 0.07 | <10^-6^ |
| rs1999478 | C | 0.86 | 0.07 | <10^-6^ |
| rs1330403 | G | 0.87 | 0.08 | <10^-6^ |
| rs1330403 | A | 0.62 | 0.06 | <10^-6^ |

| **Table S2.** Association between intake of *AMY1*-GRS and CVD and mortality risk (n=20,264) ^a^. | | | | |  |  |
| --- | --- | --- | --- | --- | --- | --- |
|  | Quintiles of *AMY1*-GRS ^b^ | | | | |  |
|  | Quintile 1 | Quintile 2 | Quintile 3 | Quintile 4 | Quintile 5 | *P* for trend |
| **Participants** | 4,101 | 4,843 | 3,242 | 4,138 | 3,940 |  |
| ***AMY1* copy number (mean)** | 5.15 | 6.37 | 6.54 | 7.31 | 8.28 |  |
| **CVD** | 1 [Reference] | 0.95 (0.87, 1.04) | 1.06 (0.96, 1.18) | 1.02 (0.93, 1.12) | 1.02 (0.93, 1.13) | 0.25 |
| **CHD** | 1 [Reference] | 1.06 (0.94, 1.20) | 1.06 (0.93, 1.21) | 1.04 (0.92, 1.18) | 1.09 (0.96, 1.24) | 0.31 |
| **Ischemic stroke** | 1 [Reference] | 0.85 (0.74, 0.98) | 1.09 (0.93, 1.28) | 0.98 (0.85, 1.14) | 0.98 (0.84, 1.13) | 0.47 |
| **All-cause mortality** | 1 [Reference] | 1.02 (0.95, 1.09) | 1.11 (1.03, 1.20) | 1.02 (0.95, 1.10) | 1.03 (0.96, 1.11) | 0.34 |
| **CVD mortality** | 1 [Reference] | 0.96 (0.86, 1.09) | 1.06 (0.93, 1.21) | 1.01 (0.89, 1.14) | 1.05 (0.93, 1.19) | 0.31 |
| CVD, cardiovascular disease; CHD, coronary heart disease. | | | | | | |
| ^a^ Obtained by using multiple linear regression. | | | | | | |
| ^b^ Adjusted for age, and sex. | | | | | | |

| **Table S3.** Association between starch intake and plasma proteins (n=3,680) ^a^. | | | | | | | | | |  |  |  |
| --- | --- | --- | --- | --- | --- | --- | --- | --- | --- | --- | --- | --- |
| **Proteins** | **Model 1** | | | | **Model 2** | | | | **Model 3** | | | |
|  | **Beta** | ***P* value** | **95% CI** | | **Beta** | ***P* value** | **95% CI** | | **Beta** | ***P* value** | **95% CI** | |
| IL8 | -0.08504 | 0.22227 | -0.22161 | 0.05154 | -0.02326 | 0.73953 | -0.16039 | 0.11388 | -0.01595 | 0.81878 | -0.15242 | 0.12052 |
| VEGFA | -0.24705 | 0.00037 | -0.38293 | -0.11117 | -0.19990 | 0.00404 | -0.33615 | -0.06365 | -0.14698 | 0.03524 | -0.28379 | -0.01017 |
| AM | -0.33874 | 0.00000 | -0.47784 | -0.19964 | -0.29270 | 0.00004 | -0.43204 | -0.15335 | -0.19240 | 0.00875 | -0.33620 | -0.04860 |
| CD40L | -0.07688 | 0.26336 | -0.21162 | 0.05786 | -0.04451 | 0.51600 | -0.17883 | 0.08982 | -0.03081 | 0.65154 | -0.16455 | 0.10293 |
| GDF15 | -0.34190 | 0.00000 | -0.48592 | -0.19787 | -0.21877 | 0.00442 | -0.36936 | -0.06818 | -0.18895 | 0.01369 | -0.33916 | -0.03875 |
| PlGF | -0.09843 | 0.17101 | -0.23938 | 0.04251 | -0.07061 | 0.32539 | -0.21137 | 0.07014 | -0.02934 | 0.68271 | -0.17004 | 0.11137 |
| SELE | -0.16122 | 0.02172 | -0.29888 | -0.02356 | -0.13758 | 0.04930 | -0.27475 | -0.00042 | -0.05776 | 0.41566 | -0.19688 | 0.08135 |
| EGF | 0.00589 | 0.93171 | -0.12881 | 0.14059 | 0.03579 | 0.60082 | -0.09831 | 0.16988 | 0.04822 | 0.47883 | -0.08526 | 0.18170 |
| OPG | -0.02828 | 0.69121 | -0.16786 | 0.11130 | 0.00236 | 0.97349 | -0.13704 | 0.14177 | 0.00929 | 0.89560 | -0.12944 | 0.14802 |
| SRC | 0.05363 | 0.43896 | -0.08221 | 0.18947 | 0.03150 | 0.64765 | -0.10363 | 0.16664 | 0.02532 | 0.71203 | -0.10916 | 0.15980 |
| IL1ra | -0.29480 | 0.00006 | -0.43880 | -0.15079 | -0.30789 | 0.00003 | -0.45175 | -0.16403 | -0.18739 | 0.01605 | -0.33991 | -0.03487 |
| IL6 | -0.32384 | 0.00000 | -0.46014 | -0.18754 | -0.24039 | 0.00068 | -0.37905 | -0.10174 | -0.17173 | 0.01631 | -0.31184 | -0.03162 |
| CSTB | -0.21456 | 0.00225 | -0.35215 | -0.07698 | -0.15931 | 0.02420 | -0.29784 | -0.02079 | -0.08564 | 0.23079 | -0.22573 | 0.05445 |
| MCP1 | -0.11976 | 0.08634 | -0.25664 | 0.01711 | -0.08888 | 0.20196 | -0.22542 | 0.04766 | -0.05764 | 0.40692 | -0.19389 | 0.07861 |
| KLK6 | 0.07671 | 0.26855 | -0.05920 | 0.21261 | 0.04932 | 0.47578 | -0.08626 | 0.18490 | 0.01216 | 0.86031 | -0.12328 | 0.14760 |
| Gal3 | 0.04456 | 0.52638 | -0.09333 | 0.18246 | 0.02170 | 0.75687 | -0.11573 | 0.15914 | 0.05475 | 0.43386 | -0.08240 | 0.19190 |
| PAR1 | -0.07017 | 0.30963 | -0.20557 | 0.06523 | -0.05119 | 0.45627 | -0.18589 | 0.08351 | -0.04053 | 0.55348 | -0.17461 | 0.09355 |
| TRAIL | -0.10271 | 0.13788 | -0.23840 | 0.03298 | -0.06557 | 0.34274 | -0.20104 | 0.06991 | -0.01414 | 0.83825 | -0.14998 | 0.12169 |
| hK11 | -0.04612 | 0.50747 | -0.18255 | 0.09031 | -0.03598 | 0.60293 | -0.17159 | 0.09962 | -0.05675 | 0.41015 | -0.19184 | 0.07833 |
| TIE2 | 0.03105 | 0.65458 | -0.10502 | 0.16713 | 0.02158 | 0.75460 | -0.11379 | 0.15696 | 0.05308 | 0.44105 | -0.08198 | 0.18814 |
| TF | -0.02739 | 0.69613 | -0.16490 | 0.11011 | -0.02528 | 0.71716 | -0.16206 | 0.11151 | -0.03890 | 0.57547 | -0.17507 | 0.09727 |
| TNFR1 | -0.11420 | 0.10740 | -0.25323 | 0.02483 | -0.09259 | 0.19332 | -0.23212 | 0.04694 | -0.02070 | 0.77318 | -0.16154 | 0.12013 |
| PDGFsub | -0.01472 | 0.83086 | -0.14987 | 0.12042 | 0.01955 | 0.77598 | -0.11512 | 0.15422 | 0.03768 | 0.58182 | -0.09644 | 0.17180 |
| IL27A | 0.18778 | 0.00673 | 0.05199 | 0.32356 | 0.12742 | 0.06675 | -0.00880 | 0.26365 | 0.11709 | 0.09053 | -0.01850 | 0.25269 |
| CSF1 | -0.17168 | 0.01333 | -0.30762 | -0.03574 | -0.16265 | 0.01916 | -0.29873 | -0.02657 | -0.10675 | 0.12595 | -0.24349 | 0.02999 |
| CXCL1 | 0.00211 | 0.97555 | -0.13261 | 0.13682 | 0.03985 | 0.56112 | -0.09457 | 0.17427 | 0.04969 | 0.46653 | -0.08409 | 0.18347 |
| LOX1 | -0.05744 | 0.40552 | -0.19280 | 0.07793 | 0.03398 | 0.62900 | -0.10391 | 0.17188 | 0.04758 | 0.49686 | -0.08970 | 0.18485 |
| TRAILR2 | -0.27849 | 0.00008 | -0.41629 | -0.14070 | -0.19203 | 0.00774 | -0.33332 | -0.05074 | -0.14717 | 0.04141 | -0.28860 | -0.00573 |
| FGF23 | -0.13131 | 0.05616 | -0.26608 | 0.00345 | -0.11744 | 0.08605 | -0.25155 | 0.01666 | -0.06765 | 0.32399 | -0.20212 | 0.06681 |
| SCF | 0.27151 | 0.00009 | 0.13620 | 0.40683 | 0.17448 | 0.01340 | 0.03622 | 0.31274 | 0.13160 | 0.06227 | -0.00675 | 0.26995 |
| IL18 | -0.11571 | 0.10242 | -0.25457 | 0.02316 | -0.08510 | 0.22988 | -0.22404 | 0.05384 | -0.03527 | 0.61938 | -0.17449 | 0.10394 |
| IL6RA | -0.05994 | 0.38422 | -0.19499 | 0.07510 | -0.06533 | 0.34114 | -0.19988 | 0.06921 | -0.03399 | 0.61970 | -0.16826 | 0.10028 |
| TNFR2 | -0.05455 | 0.43666 | -0.19204 | 0.08294 | -0.03164 | 0.65371 | -0.16991 | 0.10663 | 0.01683 | 0.81164 | -0.12162 | 0.15529 |
| MMP3 | -0.03789 | 0.65733 | -0.20536 | 0.12958 | -0.03018 | 0.72254 | -0.19680 | 0.13644 | -0.03735 | 0.65885 | -0.20318 | 0.12849 |
| HSP27 | 0.04440 | 0.51930 | -0.09067 | 0.17948 | 0.04093 | 0.55014 | -0.09335 | 0.17520 | 0.05880 | 0.38866 | -0.07492 | 0.19252 |
| TNFSF14 | -0.11872 | 0.08454 | -0.25363 | 0.01619 | -0.07132 | 0.30053 | -0.20635 | 0.06372 | -0.02672 | 0.69833 | -0.16187 | 0.10843 |
| PRL | 0.03815 | 0.58612 | -0.09922 | 0.17553 | -0.00235 | 0.97325 | -0.13955 | 0.13486 | -0.01405 | 0.84012 | -0.15062 | 0.12251 |
| MPO | -0.05508 | 0.42539 | -0.19054 | 0.08038 | -0.04026 | 0.56010 | -0.17573 | 0.09520 | -0.00576 | 0.93343 | -0.14101 | 0.12948 |
| GH | -0.09955 | 0.23695 | -0.26455 | 0.06546 | -0.03627 | 0.66681 | -0.20141 | 0.12888 | -0.13363 | 0.11680 | -0.30065 | 0.03338 |
| MMP1 | -0.02129 | 0.75808 | -0.15681 | 0.11423 | 0.05760 | 0.40793 | -0.07884 | 0.19404 | 0.05668 | 0.41311 | -0.07909 | 0.19244 |
| RETN | -0.07692 | 0.26443 | -0.21204 | 0.05820 | -0.05714 | 0.40712 | -0.19227 | 0.07799 | -0.03612 | 0.59893 | -0.17075 | 0.09851 |
| FAS | -0.16123 | 0.02241 | -0.29961 | -0.02285 | -0.13222 | 0.06135 | -0.27073 | 0.00629 | -0.08103 | 0.25276 | -0.21992 | 0.05786 |
| PAPPA | 0.04894 | 0.51250 | -0.09755 | 0.19542 | 0.02530 | 0.73367 | -0.12050 | 0.17111 | 0.03171 | 0.66827 | -0.11336 | 0.17677 |
| PTX3 | 0.01882 | 0.79051 | -0.12004 | 0.15767 | 0.01851 | 0.79286 | -0.11967 | 0.15668 | -0.00198 | 0.97744 | -0.13963 | 0.13566 |
| REN | -0.02420 | 0.73508 | -0.16439 | 0.11600 | 0.03975 | 0.58045 | -0.10124 | 0.18075 | 0.06125 | 0.39259 | -0.07920 | 0.20170 |
| CHI3L1 | -0.11848 | 0.09729 | -0.25853 | 0.02158 | -0.06755 | 0.34519 | -0.20783 | 0.07273 | -0.03197 | 0.65455 | -0.17206 | 0.10811 |
| ST2 | -0.04827 | 0.51513 | -0.19365 | 0.09711 | -0.09195 | 0.21390 | -0.23696 | 0.05307 | -0.07665 | 0.29809 | -0.22104 | 0.06775 |
| TIM | -0.30413 | 0.00002 | -0.44479 | -0.16347 | -0.23222 | 0.00140 | -0.37465 | -0.08979 | -0.19195 | 0.00827 | -0.33438 | -0.04953 |
| mAmP | -0.01481 | 0.84591 | -0.16427 | 0.13464 | -0.00883 | 0.90747 | -0.15772 | 0.14007 | -0.02860 | 0.70565 | -0.17705 | 0.11986 |
| TRANCE | -0.02301 | 0.74013 | -0.15903 | 0.11300 | -0.05263 | 0.44670 | -0.18823 | 0.08297 | 0.00988 | 0.88698 | -0.12646 | 0.14623 |
| HGF | -0.23185 | 0.00108 | -0.37081 | -0.09290 | -0.16309 | 0.02385 | -0.30455 | -0.02164 | -0.05997 | 0.41794 | -0.20511 | 0.08517 |
| PSGL1 | -0.11043 | 0.15449 | -0.26247 | 0.04160 | -0.10983 | 0.15497 | -0.26120 | 0.04155 | -0.08236 | 0.28466 | -0.23327 | 0.06855 |
| MB | 0.15284 | 0.03850 | 0.00810 | 0.29758 | 0.08656 | 0.24269 | -0.05868 | 0.23180 | 0.13610 | 0.06636 | -0.00920 | 0.28140 |
| TM | 0.04232 | 0.55058 | -0.09667 | 0.18131 | 0.02779 | 0.69457 | -0.11097 | 0.16656 | 0.06005 | 0.39515 | -0.07839 | 0.19849 |
| IL16 | 0.01378 | 0.84362 | -0.12321 | 0.15078 | 0.00432 | 0.95058 | -0.13241 | 0.14106 | 0.07140 | 0.30926 | -0.06626 | 0.20907 |
| MMP10 | -0.22539 | 0.00105 | -0.36014 | -0.09065 | -0.11743 | 0.09484 | -0.25523 | 0.02037 | -0.15361 | 0.02861 | -0.29115 | -0.01607 |
| UPAR | -0.17626 | 0.01127 | -0.31256 | -0.03996 | -0.01848 | 0.80261 | -0.16337 | 0.12642 | 0.00848 | 0.90840 | -0.13596 | 0.15291 |
| CCL4 | 0.04558 | 0.51238 | -0.09082 | 0.18198 | 0.05664 | 0.41363 | -0.07917 | 0.19245 | 0.09335 | 0.17720 | -0.04226 | 0.22896 |
| CTSD | -0.25929 | 0.00029 | -0.39952 | -0.11905 | -0.21358 | 0.00295 | -0.35432 | -0.07283 | -0.12080 | 0.09977 | -0.26466 | 0.02305 |
| RAGE | 0.02881 | 0.67664 | -0.10664 | 0.16426 | 0.03237 | 0.63859 | -0.10274 | 0.16747 | -0.02926 | 0.67287 | -0.16512 | 0.10660 |
| CCL3 | -0.04337 | 0.54002 | -0.18212 | 0.09538 | -0.04882 | 0.48951 | -0.18732 | 0.08967 | 0.02002 | 0.77852 | -0.11954 | 0.15959 |
| MMP7 | -0.19950 | 0.00442 | -0.33682 | -0.06218 | -0.12732 | 0.07147 | -0.26577 | 0.01113 | -0.10604 | 0.13184 | -0.24398 | 0.03190 |
| CXCL6 | -0.03317 | 0.62939 | -0.16794 | 0.10159 | -0.04327 | 0.52689 | -0.17731 | 0.09078 | -0.02008 | 0.76820 | -0.15368 | 0.11351 |
| ITGB1BP | 0.04350 | 0.53613 | -0.09435 | 0.18136 | 0.06137 | 0.38046 | -0.07581 | 0.19855 | 0.06839 | 0.32615 | -0.06815 | 0.20494 |
| CXCL16 | -0.10281 | 0.13751 | -0.23851 | 0.03289 | -0.05456 | 0.43076 | -0.19033 | 0.08120 | -0.02904 | 0.67398 | -0.16439 | 0.10630 |
| Dkk1 | -0.01911 | 0.78134 | -0.15407 | 0.11585 | -0.01234 | 0.85697 | -0.14660 | 0.12191 | 0.00002 | 0.99971 | -0.13362 | 0.13367 |
| SIRT2 | -0.01567 | 0.81957 | -0.15031 | 0.11897 | -0.00589 | 0.93129 | -0.13978 | 0.12800 | 0.01040 | 0.87850 | -0.12293 | 0.14372 |
| GAL | 0.22944 | 0.00122 | 0.09051 | 0.36837 | 0.14100 | 0.04943 | 0.00035 | 0.28164 | 0.07833 | 0.27796 | -0.06320 | 0.21986 |
| AGRP | 0.00067 | 0.99227 | -0.13532 | 0.13667 | -0.01155 | 0.86711 | -0.14682 | 0.12373 | -0.03627 | 0.59794 | -0.17109 | 0.09856 |
| CD40 | -0.03280 | 0.63310 | -0.16750 | 0.10190 | -0.03541 | 0.60457 | -0.16945 | 0.09864 | -0.00890 | 0.89608 | -0.14255 | 0.12475 |
| tPA | -0.09597 | 0.17528 | -0.23477 | 0.04282 | -0.10059 | 0.15372 | -0.23881 | 0.03763 | -0.01102 | 0.87797 | -0.15169 | 0.12966 |
| HBEGF | 0.01351 | 0.84460 | -0.12162 | 0.14864 | 0.00991 | 0.88496 | -0.12443 | 0.14426 | 0.03212 | 0.63810 | -0.10174 | 0.16597 |
| ESM1 | 0.21039 | 0.00255 | 0.07378 | 0.34701 | 0.14778 | 0.03492 | 0.01047 | 0.28509 | 0.10548 | 0.13230 | -0.03189 | 0.24286 |
| VEGFD | -0.07431 | 0.28573 | -0.21078 | 0.06215 | 0.01051 | 0.88118 | -0.12731 | 0.14833 | -0.04716 | 0.50395 | -0.18549 | 0.09118 |
| MMP12 | -0.11998 | 0.09329 | -0.26010 | 0.02014 | 0.03118 | 0.67746 | -0.11577 | 0.17812 | 0.03547 | 0.63438 | -0.11075 | 0.18168 |
| SPON1 | -0.04102 | 0.55888 | -0.17859 | 0.09655 | -0.00269 | 0.96943 | -0.14043 | 0.13505 | -0.00248 | 0.97172 | -0.13953 | 0.13458 |
| CASP8 | 0.00937 | 0.89557 | -0.13054 | 0.14927 | 0.02812 | 0.69220 | -0.11113 | 0.16737 | 0.05811 | 0.41213 | -0.08079 | 0.19702 |
| CTSL1 | -0.08821 | 0.21216 | -0.22681 | 0.05039 | -0.06614 | 0.34994 | -0.20486 | 0.07258 | -0.03073 | 0.66361 | -0.16924 | 0.10778 |
| CX3CL1 | 0.02930 | 0.67212 | -0.10641 | 0.16501 | 0.01003 | 0.88424 | -0.12502 | 0.14508 | 0.00795 | 0.90766 | -0.12643 | 0.14233 |
| FABP4 | -0.36916 | 0.00000 | -0.51491 | -0.22341 | -0.39455 | 0.00000 | -0.54016 | -0.24894 | -0.23626 | 0.00455 | -0.39941 | -0.07310 |
| LEP | -0.41050 | 0.00000 | -0.57625 | -0.24474 | -0.49870 | 0.00000 | -0.66561 | -0.33179 | -0.29605 | 0.00669 | -0.50995 | -0.08216 |
| CCL20 | -0.30903 | 0.00001 | -0.44353 | -0.17453 | -0.25121 | 0.00028 | -0.38652 | -0.11590 | -0.20827 | 0.00260 | -0.34377 | -0.07278 |
| CA125 | 0.04369 | 0.54566 | -0.09805 | 0.18543 | 0.02101 | 0.77012 | -0.11998 | 0.16201 | 0.04136 | 0.56401 | -0.09920 | 0.18193 |
| NEMO | 0.02548 | 0.71475 | -0.11121 | 0.16218 | 0.00746 | 0.91436 | -0.12852 | 0.14344 | 0.02611 | 0.70546 | -0.10933 | 0.16155 |
| FS | -0.09535 | 0.17313 | -0.23257 | 0.04186 | -0.06221 | 0.37276 | -0.19905 | 0.07462 | -0.01709 | 0.80672 | -0.15403 | 0.11985 |
| PECAM1 | 0.06710 | 0.33231 | -0.06858 | 0.20277 | 0.05077 | 0.46100 | -0.08424 | 0.18577 | 0.07974 | 0.24564 | -0.05489 | 0.21436 |
| NTproBN | 0.07530 | 0.33401 | -0.07750 | 0.22809 | 0.07970 | 0.30246 | -0.07182 | 0.23123 | 0.06001 | 0.43594 | -0.09101 | 0.21103 |
| ECP | 0.07674 | 0.26750 | -0.05893 | 0.21242 | 0.06564 | 0.34088 | -0.06947 | 0.20075 | 0.10695 | 0.12052 | -0.02808 | 0.24197 |
| CI, confidence interval. | | | | | | | | | |  |  |  |
| ^a^ Using multiple linear regression analysis. Proteins were standardized as z-scores and corrected *p*<0.05/88. | | | | | | | | | |  |  |  |
| Model 1 was adjusted for age, sex, season, and total energy intake. | | | | | | | | | |  |  |  |
| Model 2 was adjusted for age, sex, season, total energy intake, educational level, smoking status, alcohol consumption, and physical activity. | | | | | | | | | | | | |
| Model 3 was further adjusted for body mass index. | | | | | | | | | |  |  |  |

| **Table S4.** Association between plasma protein and CVD and mortality risk (n=3,680) ^a^. | | | | | | | | | | |
| --- | --- | --- | --- | --- | --- | --- | --- | --- | --- | --- |
| **Proteins** | **CVD** | | **CHD** | | **Ischemic stroke** | | **All cause mortality** | | **CVD mortality** | |
|  | HR (95% CI) | *P* value | HR (95% CI) | *P* value | HR (95% CI) | *P* value | HR (95% CI) | *P* value | HR (95% CI) | *P* value |
| **Leptin** |  |  |  |  |  |  |  |  |  |  |
| Model 1 | 1.10 (1.00, 1.21) | 0.04 | 1.09 (0.96, 1.23) | 0.18 | 1.15 (1.00, 1.33) | 0.05 | 1.09 (1.02, 1.16) | 0.02 | 1.25 (1.10, 1.42) | <0.001 |
| Model 2 | 1.13 (1.03, 1.24) | 0.01 | 1.14 (1.00, 1.29) | 0.04 | 1.17 (1.01, 1.34) | 0.04 | 1.14 (1.06, 1.22) | <0.001 | 1.29 (1.13, 1.47) | <0.001 |
| Model 3 | 1.09 (0.97, 1.23) | 0.14 | 1.02 (0.87, 1.20) | 0.78 | 1.18 (0.98, 1.41) | 0.08 | 1.02 (0.94, 1.11) | 0.63 | 1.07 (0.91, 1.26) | 0.43 |
| **FABP4** |  |  |  |  |  |  |  |  |  |  |
| Model 1 | 1.11 (1.02, 1.20) | 0.02 | 1.12 (1.00, 1.24) | 0.05 | 1.11 (0.98, 1.25) | 0.10 | 1.13 (1.07, 1.20) | <0.001 | 1.29 (1.15, 1.44) | <0.001 |
| Model 2 | 1.10 (1.02, 1.20) | 0.02 | 1.12 (1.01, 1.25) | 0.03 | 1.10 (0.97, 1.24) | 0.14 | 1.14 (1.07, 1.21) | <0.001 | 1.29 (1.15, 1.44) | <0.001 |
| Model 3 | 1.07 (0.98, 1.17) | 0.14 | 1.06 (0.94, 1.19) | 0.38 | 1.08 (0.94, 1.23) | 0.28 | 1.08 (1.01, 1.15) | 0.03 | 1.15 (1.02, 1.31) | 0.02 |
| **IL1ra** |  |  |  |  |  |  |  |  |  |  |
| Model 1 | 1.15 (1.06, 1.23) | <0.001 | 1.15 (1.04, 1.27) | 0.01 | 1.16 (1.04, 1.30) | 0.01 | 1.19 (1.13, 1.25) | <0.001 | 1.19 (1.08, 1.31) | <0.001 |
| Model 2 | 1.14 (1.05, 1.22) | <0.01 | 1.14 (1.03, 1.27) | 0.01 | 1.15 (1.03, 1.29) | 0.01 | 1.19 (1.13, 1.25) | <0.001 | 1.18 (1.07, 1.31) | <0.001 |
| Model 3 | 1.12 (1.04, 1.22) | <0.01 | 1.11 (1.00, 1.24) | 0.06 | 1.15 (1.02, 1.29) | 0.02 | 1.15 (1.09, 1.22) | <0.001 | 1.09 (0.98, 1.21) | 0.13 |
| **CCL20** |  |  |  |  |  |  |  |  |  |  |
| Model 1 | 1.11 (1.04, 1.19) | <0.01 | 1.15 (1.05, 1.25) | <0.01 | 1.11 (1.00, 1.23) | 0.05 | 1.17 (1.11, 1.23) | <0.001 | 1.12 (1.01, 1.23) | 0.02 |
| Model 2 | 1.09 (1.01, 1.17) | 0.02 | 1.12 (1.02, 1.23) | 0.01 | 1.09 (0.98, 1.21) | 0.12 | 1.13 (1.07, 1.19) | <0.001 | 1.09 (0.98, 1.20) | 0.11 |
| Model 3 | 1.08 (1.00, 1.16) | 0.04 | 1.11 (1.01, 1.22) | 0.03 | 1.08 (0.97, 1.21) | 0.15 | 1.11 (1.06, 1.17) | <0.001 | 1.05 (0.95, 1.17) | 0.34 |
| **Adrenomedullin** |  |  |  |  |  |  |  |  |  |  |
| Model 1 | 1.10 (1.02, 1.19) | 0.02 | 1.13 (1.02, 1.26) | 0.02 | 1.20 (1.06, 1.35) | <0.01 | 1.14 (1.08, 1.21) | <0.001 | 1.09 (0.98, 1.22) | 0.10 |
| Model 2 | 1.08 (1.00, 1.17) | 0.05 | 1.12 (1.00, 1.24) | 0.04 | 1.18 (1.04, 1.33) | 0.01 | 1.09 (1.03, 1.16) | <0.01 | 1.06 (0.95, 1.18) | 0.29 |
| Model 3 | 1.06 (0.98, 1.15) | 0.15 | 1.08 (0.97, 1.20) | 0.19 | 1.17 (1.03, 1.32) | 0.02 | 1.06 (1.00, 1.12) | 0.07 | 0.99 (0.89, 1.10) | 0.83 |
| CI, confidence interval; CCL20, C-C motif chemokine 20; CVD, cardiovascular disease; CHD, coronary heart disease; FABP4, fatty acid-binding protein; HR, hazard ratio; IL1ra, interleukin-1 receptor antagonist protein. | | | | | | | | | | |
| ^a^ Values are hazard ratios (95% confidence interval) for per one standard deviation increase unless otherwise indicated. | | | | | | | | | | |
| Model 1 was adjusted age and sex. | | | | | | | | | | |
| Model 2 was adjusted for model 1 plus physical activity, smoking status, alcohol consumption, educational level, and heredity scores (including cancer, infarct, stroke, and diabetes). | | | | | | | | | | |
| Model 3 was adjusted for model 2 plus body mass index. | | | | | | |  |  |  |  |

| **Table S5.** Association between *AMY1* copy number and plasma proteins (n=3,254). | | | | |
| --- | --- | --- | --- | --- |
| **Proteins** | **Beta** | ***P* value** | **95% CI** | |
| IL8 | 0.01186343 | 0.499434942 | -0.022573457 | 0.046300314 |
| VEGFA | 0.02091537 | 0.236469471 | -0.013718079 | 0.055548818 |
| AM | -0.00928069 | 0.60845755 | -0.044798291 | 0.026236909 |
| CD40L | 0.01553564 | 0.372474947 | -0.018614201 | 0.049685484 |
| GDF15 | 0.00217695 | 0.906877426 | -0.034308482 | 0.038662377 |
| PlGF | 0.0055245 | 0.762329046 | -0.030289295 | 0.041338291 |
| SELE | -0.00998245 | 0.578457784 | -0.045204183 | 0.025239282 |
| EGF | 0.03072579 | 0.076033809 | -0.003219325 | 0.064670898 |
| OPG | 0.00063686 | 0.971956536 | -0.034880559 | 0.036154285 |
| SRC | 0.00748769 | 0.664681797 | -0.026378845 | 0.041354231 |
| IL1ra | -0.01115006 | 0.548635173 | -0.047596048 | 0.025295928 |
| IL6 | -0.02833217 | 0.111475251 | -0.063225184 | 0.006560845 |
| CSTB | 0.01243537 | 0.482817687 | -0.022303764 | 0.047174498 |
| MCP1 | -0.00142495 | 0.93487868 | -0.035615732 | 0.032765836 |
| KLK6 | 0.00342394 | 0.845747228 | -0.031080562 | 0.037928443 |
| Gal3 | 0.02368668 | 0.186888569 | -0.011493759 | 0.058867112 |
| PAR1 | 0.01795989 | 0.301373557 | -0.01610737 | 0.052027153 |
| TRAIL | 0.00971373 | 0.580259897 | -0.024723083 | 0.044150534 |
| hK11 | -0.01203166 | 0.499358115 | -0.046950635 | 0.022887311 |
| TIE2 | -0.00608673 | 0.72952325 | -0.040599702 | 0.028426234 |
| TF | 0.0015575 | 0.930513689 | -0.033460697 | 0.036575688 |
| TNFR1 | 0.01220568 | 0.497068519 | -0.023030047 | 0.047441405 |
| PDGFsub | 0.02029818 | 0.24099748 | -0.013639001 | 0.054235353 |
| IL27A | -0.00532006 | 0.760666957 | -0.039561545 | 0.028921429 |
| CSF1 | 0.00299289 | 0.865220045 | -0.031577134 | 0.037562914 |
| CXCL1 | 0.00136065 | 0.936822552 | -0.032294049 | 0.035015347 |
| LOX1 | -0.01673963 | 0.336047213 | -0.050852193 | 0.017372923 |
| TRAILR2 | 0.01234252 | 0.481304873 | -0.022018365 | 0.046703401 |
| FGF23 | 0.00435791 | 0.801214456 | -0.02957641 | 0.038292238 |
| SCF | 0.00848641 | 0.629160283 | -0.025966605 | 0.042939423 |
| IL18 | -0.03282392 | 0.066422871 | -0.067873751 | 0.00222591 |
| IL6RA | 0.01806163 | 0.301148404 | -0.016182658 | 0.05230592 |
| TNFR2 | -0.00572892 | 0.74722366 | -0.040577278 | 0.029119437 |
| MMP3 | 0.02951761 | 0.172304477 | -0.012877508 | 0.071912733 |
| HSP27 | 0.01780083 | 0.303383581 | -0.016105411 | 0.05170707 |
| TNFSF14 | 0.02351126 | 0.174845452 | -0.010457191 | 0.057479713 |
| PRL | 0.00866022 | 0.625976602 | -0.026174641 | 0.043495078 |
| MPO | -0.00566111 | 0.745329708 | -0.039831781 | 0.028509566 |
| GH | 0.03250378 | 0.125814703 | -0.009116868 | 0.074124434 |
| MMP1 | 0.01260572 | 0.467396167 | -0.021400475 | 0.046611905 |
| RETN | 0.01961457 | 0.261782863 | -0.014650205 | 0.053879349 |
| FAS | 0.01110703 | 0.531271116 | -0.023673856 | 0.045887918 |
| PAPPA | -0.00033496 | 0.98576646 | -0.037146188 | 0.036476267 |
| PTX3 | -0.0157567 | 0.37602915 | -0.050651604 | 0.019138212 |
| REN | 0.00058176 | 0.974411089 | -0.034975731 | 0.036139243 |
| CHI3L1 | 0.01108375 | 0.536225953 | -0.024047194 | 0.046214693 |
| ST2 | -0.01878281 | 0.318553557 | -0.055699224 | 0.018133596 |
| TIM | -0.00675152 | 0.711444494 | -0.042533943 | 0.029030895 |
| mAmP | -0.01682042 | 0.389437452 | -0.055137947 | 0.021497104 |
| TRANCE | -0.01828053 | 0.294970703 | -0.052499496 | 0.015938434 |
| HGF | 0.00943901 | 0.59766483 | -0.02562453 | 0.044502547 |
| PSGL1 | -0.02401013 | 0.220476552 | -0.062426411 | 0.014406158 |
| MB | -0.00881204 | 0.636331871 | -0.045349399 | 0.027725316 |
| TM | 0.00939484 | 0.602822087 | -0.026001962 | 0.044791636 |
| IL16 | -0.00925994 | 0.601331415 | -0.044005548 | 0.025485676 |
| MMP10 | -0.00455932 | 0.792056612 | -0.038464097 | 0.029345466 |
| UPAR | 0.00010631 | 0.995190834 | -0.034473673 | 0.034686297 |
| CCL4 | -0.01668214 | 0.342038275 | -0.051101899 | 0.017737622 |
| CTSD | 0.01090397 | 0.546996105 | -0.024590409 | 0.046398349 |
| RAGE | 0.00194654 | 0.910961693 | -0.032180476 | 0.036073557 |
| CCL3 | -0.02474433 | 0.16944164 | -0.060046664 | 0.010557994 |
| MMP7 | -0.02684306 | 0.128069541 | -0.061419894 | 0.007733784 |
| CXCL6 | 0.00270492 | 0.877148237 | -0.031600429 | 0.037010269 |
| ITGB1BP | 0.02552403 | 0.14826379 | -0.009084444 | 0.060132498 |
| CXCL16 | 0.01041157 | 0.549962672 | -0.023732371 | 0.044555511 |
| Dkk1 | 0.02495213 | 0.152218768 | -0.009210553 | 0.059114812 |
| SIRT2 | 0.02207061 | 0.202401079 | -0.011869616 | 0.056010845 |
| GAL | 0.00112481 | 0.950013449 | -0.034052017 | 0.03630163 |
| AGRP | -0.0183616 | 0.299623182 | -0.05306493 | 0.016341723 |
| CD40 | 0.03748056 | 0.031762567 | 0.003273297 | 0.071687818 |
| tPA | 0.02265364 | 0.204792442 | -0.012367975 | 0.057675251 |
| HBEGF | 0.02303316 | 0.186485021 | -0.011145319 | 0.057211638 |
| ESM1 | -1.1381E-05 | 0.999485882 | -0.034640491 | 0.034617728 |
| VEGFD | 0.01422272 | 0.417576609 | -0.02017369 | 0.048619138 |
| MMP12 | -0.00549223 | 0.762631458 | -0.041143605 | 0.030159145 |
| SPON1 | 0.00327099 | 0.853711352 | -0.031508896 | 0.038050867 |
| CASP8 | 0.0145145 | 0.417646614 | -0.020592944 | 0.049621943 |
| CTSL1 | -0.00143286 | 0.936287735 | -0.036575459 | 0.033709744 |
| CX3CL1 | -0.00492452 | 0.779439187 | -0.039399373 | 0.02955033 |
| FABP4 | 0.00612357 | 0.7456461 | -0.030886518 | 0.043133652 |
| LEP | 0.00566704 | 0.791130446 | -0.036284184 | 0.047618255 |
| CCL20 | -0.01749471 | 0.307889987 | -0.051129852 | 0.016140434 |
| CA125 | -0.01830713 | 0.313347223 | -0.053904194 | 0.017289925 |
| NEMO | 0.02403426 | 0.167110176 | -0.010067525 | 0.058136039 |
| FS | 0.00448469 | 0.799417613 | -0.030117229 | 0.039086601 |
| PECAM1 | 0.03066594 | 0.082654721 | -0.00396872 | 0.065300605 |
| NTproBN | -0.02704913 | 0.170911086 | -0.065773791 | 0.011675523 |
| ECP | -0.01853979 | 0.286822274 | -0.05266266 | 0.015583075 |
| CI, confidence interval. | | | | |
| Using multiple linear regression analysis with adjustment for age and sex.  Proteins were standardized as z-scores. | | | | |

**Fig. S1.** Flowchart of participant selection from the Malmö Diet and Cancer Study.

**
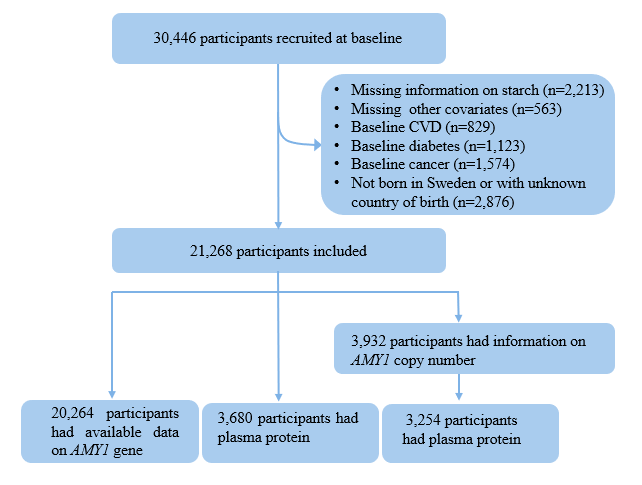
**


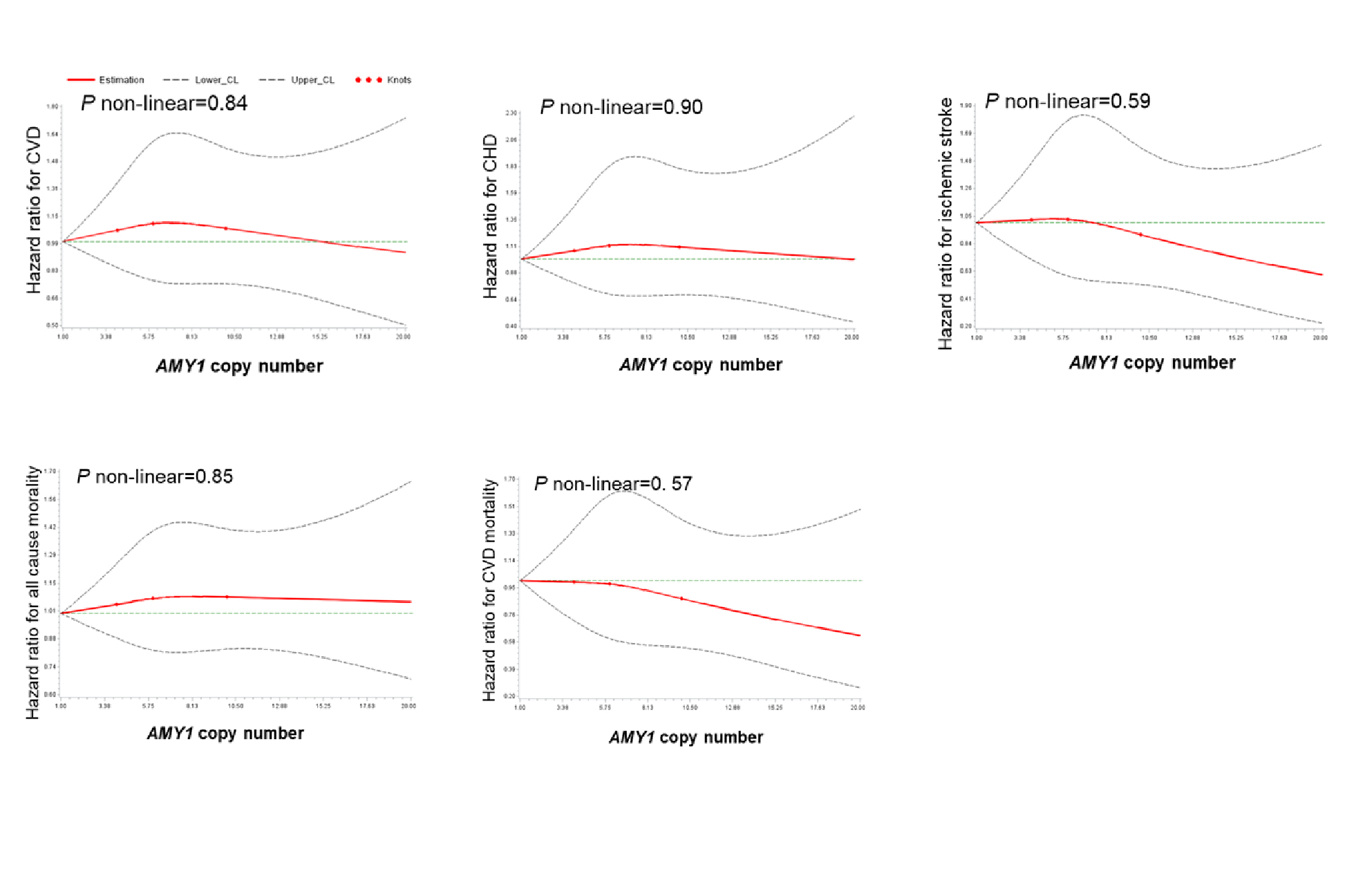
**F****ig. S2.** Restricted cubic spline plots to assess association between *AMY1* copy number and CVD and mortality.

The HRs and 95% CIs above were adjusted for age and sex.

CVD, cardiovascular disease; CHD, coronary heart disease.

**Fig. S3.** Restricted cubic spline plots to assess association between *AMY1*-GRS and CVD and mortality.

The HRs and 95% CIs above were adjusted for age and sex.

CVD, cardiovascular disease; CHD, coronary heart disease.


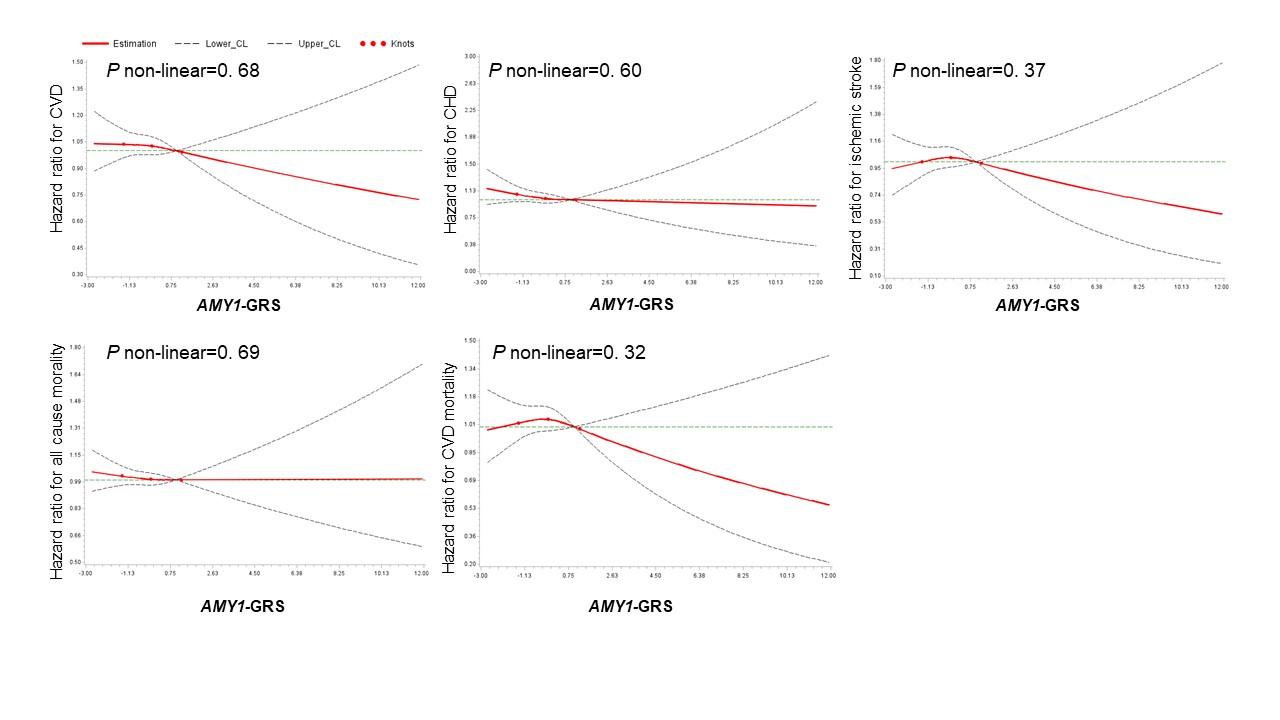
**Fig. S4.** Association between intake of starch and risk of CVD and mortality by quintiles of *AMY1*-GRS (n=20,264) ^a^.

 ^a^ The multivariable analysis was adjusted for age, sex, smoking status, drinking status, education, season, method, physical activity, heredity scores (including cancer, infarct, stroke, and diabetes), total energy intake, the modified diet index, hypertension, and body mass index.

^b^ Hazard ratio for per increase of 10% in proportion of starch energy in total energy.

CVD, cardiovascular disease; CHD, coronary heart disease; CI, confidence interval; HR, hazards ratio.
